# A machine learning ensemble to predict treatment outcomes following an Internet intervention for depression

**DOI:** 10.1017/S003329171800315X

**Published:** 2018-11-05

**Authors:** Rahel Pearson, Derek Pisner, Björn Meyer, Jason Shumake, Christopher G. Beevers

**Affiliations:** 1 Department of Psychology, Institute for Mental Health Research, University of Texas at Austin, Austin, TX, USA; 2 Gaia AG, Hamburg, Germany; 3 University of London, London, England, UK

**Keywords:** Depression treatment, Internet interventions, machine learning

## Abstract

**Background:**

Some Internet interventions are regarded as effective treatments for adult depression, but less is known about who responds to this form of treatment.

**Method:**

An elastic net and random forest were trained to predict depression symptoms and related disability after an 8-week course of an Internet intervention, Deprexis, involving adults (*N* = 283) from across the USA. Candidate predictors included psychopathology, demographics, treatment expectancies, treatment usage, and environmental context obtained from population databases. Model performance was evaluated using predictive *R*^2^ 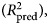 the expected variance explained in a *new* sample, estimated by 10 repetitions of 10-fold cross-validation.

**Results:**

An ensemble model was created by averaging the predictions of the elastic net and random forest. Model performance was compared with a benchmark linear autoregressive model that predicted each outcome using only its baseline. The ensemble predicted more variance in post-treatment depression (8.0% gain, 95% CI 0.8–15; total 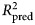
= 0.25), disability (5.0% gain, 95% CI −0.3 to 10; total 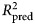
= 0.25), and well-being (11.6% gain, 95% CI 4.9–19; total 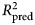
= 0.29) than the benchmark model. Important predictors included comorbid psychopathology, particularly total psychopathology and dysthymia, low symptom-related disability, treatment credibility, lower access to therapists, and time spent using certain Deprexis modules.

**Conclusion:**

A number of variables predict symptom improvement following an Internet intervention, but each of these variables makes relatively small contributions. Machine learning ensembles may be a promising statistical approach for identifying the cumulative contribution of many weak predictors to psychosocial depression treatment response.

Self-guided Internet interventions appear to effectively reduce symptoms of depression. A recent meta-analysis of 16 randomized clinical trials found that self-guided CBT interventions were significantly more effective at reducing depression symptoms than control treatments (number needed to treat = 8) (Karyotaki *et al*., 2017). However, despite the effectiveness of these treatments on average, it is clear that these interventions are neither equally effective nor effective for all participants. Identifying which individuals are most likely to benefit from a potentially effective treatment is essential to developing an efficient and personalized health care system (Cuijpers *et al*., 2016).

Precision medicine, aimed at identifying individual differences that predict beneficial and/or adverse effects, has recently emerged as a major goal in health care (Hamburg and Collins, 2010). Although many predictors of response to depression treatment using traditional statistical methods have been examined (Huang *et al*., 2014), the predictive power of each variable in isolation is often relatively weak. As a result, data-based personalized treatment recommendations are not routinely offered for any form of depression treatment (although see Fisher, 2015; Fernandez *et al*., 2017), despite the known benefits of actuarial approaches (Dawes *et al*., 1989).

New statistical methods, such as machine learning, may allow for the development of treatment algorithms that can predict with high accuracy whether or not treatment may be successful for a given individual with a specific set of attributes. Specifically, for traditional linear models to be stable and reproducible, the number of predictors must be kept small relative to the sample size, and subsets of predictors cannot be highly correlated with one another. As a consequence, traditional multiple regression is not well suited to data-mining challenges, i.e. when there are hundreds of potentially relevant predictors, many of which share at least some degree of redundancy. For such problems, machine learning methods can outperform traditional stepwise selection methods by capturing the simultaneous effect of *all* relevant predictors rather than considering discrete subsets of predictors one at a time and discarding all but the strongest (Hastie *et al*., 2009). This approach has been successfully used in medicine, most notably in oncology, where machine learning approaches have been applied to predict cancer progression and treatment outcome (Cruz and Wishart, 2007). More recently, machine learning was successfully used to predict treatment response to antidepressant medication (Khodayari-Rostamabad *et al*., 2013; Chekroud *et al*., 2016) and electroconvulsive therapy (Redlich *et al*., 2016). The current study applies machine learning methods to predict response to a psychosocial intervention for depression (for a review, see Cohen and DeRubeis, 2018).

The current study involved a secondary analysis of data from a recently published clinical trial examining the effectiveness of an Internet intervention for depression among adults recruited from across the USA (Beevers *et al*., 2017). An ensemble of elastic net and random forest learners was used to predict treatment outcomes. Symptom outcomes included interviewer-rated depression, symptom-related disability, and well-being (i.e. positive affect). Candidate predictors included relatively low-cost and easy to obtain self-report data including concurrent psychopathology, demographics, treatment expectancies, treatment usage, and, given the geographic diversity of our sample, environmental context variables mined from population databases based on participants’ postal address.

## Methods

### Study design

Data were obtained from a recently published clinical trial comparing a depression-focused Internet intervention, Deprexis, to an 8-week waitlist control condition. Treatment response was comparable for participants who received Deprexis immediately *v.* after an 8-week waiting period (Beevers *et al*., 2017). Thus, to maximize sample size for machine learning analyses, participants who received Deprexis immediately (*N* = 222) or after an 8-week waiting period (*N* = 61) were combined for analyses. Whether or not treatment was administered immediately or after a delay was also included as a predictor variable, but it was not a highly important predictor of post-treatment outcome in any model (depending on outcome and importance metric, it ranked between 67 and 114 out of 120 predictors). We used a complete case approach, utilizing data from participants who provided data at pre- and post-treatment assessments (*N* ranged from 283 to 271, depending on missing outcome data).

Deprexis is an Internet treatment for unipolar depression that was provided with relatively minimal user support. The intervention consists of 10 content modules that include behavioral activation, cognitive modification, relaxation, exercise and lifestyle modification, acceptance and mindfulness, problem-solving, childhood experiences, interpersonal skills, positive psychology, and dream work [for more detail, see Table 1 from Beevers *et al*. (2017)]. Further, daily brief messages are sent automatically by the program and are intended to remind and motivate the users to engage with the program. A recent meta-analyses of eight Deprexis trials with a total of 2402 participants has yielded a medium effect size of Hedges’ *g* = 0.54 for this intervention, compared with control conditions, with no evidence of publication bias or developer involvement bias (Twomey *et al*., 2017).

Study inclusion criteria were: (1) age between 18 and 55; (2) English fluency; (3) reliable access to the Internet (i.e. dialup or broadband access); (4) willingness to provide saliva for DNA research; (5) presence of moderate levels of depression or greater (QIDS score ⩾10) at time of eligibility screening; (6) treatment stability (no changes in psychotropic medication or psychosocial treatment in the 30 days prior to study entry); and (7) living in the USA. Exclusion criteria were: (1) presence of psychotic or substance use symptoms; (2) a diagnosis of bipolar disorder; or (3) suicidal risk (defined as having suicidal ideation with intent with/or without a plan in the last 90 days or attempting suicide in the past year).

**Fig. 1.**
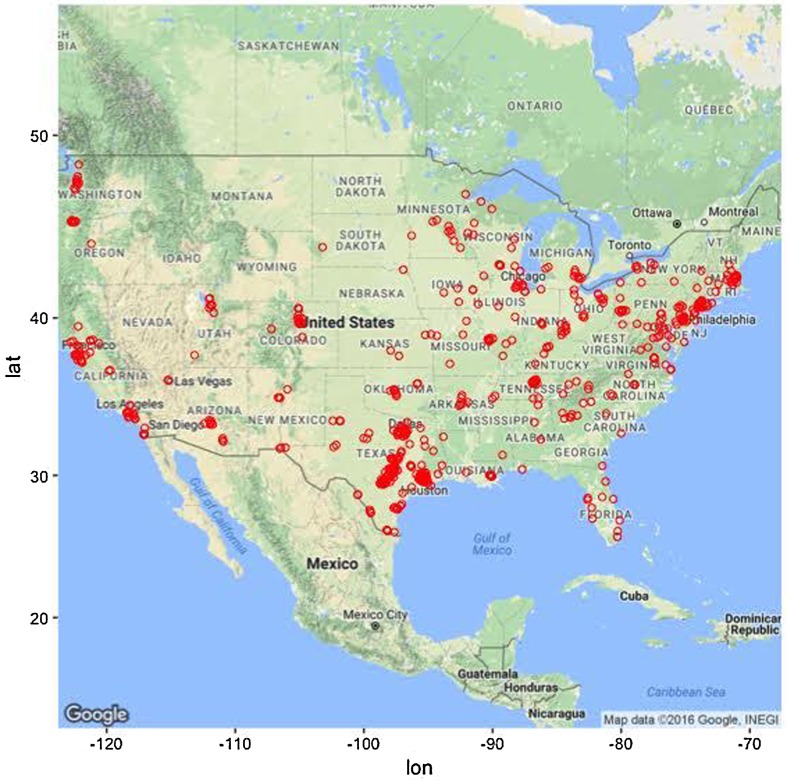
Geographical distribution of study participants. Note that most participants (approximately 50%) were recruited from the state of Texas.

Participants were primarily female (74.4%) and in their early 30s (mean age, 32 years old, range 18–55). Participants also tended to be single, non-Hispanic white, and have at least some college education. Although the majority of participants were recruited from the state of Texas (50.2%), there was a geographic diversity (see Fig. 1). Approximately 40% of the sample was currently receiving antidepressant treatment and more than 2/3 of the sample had received psychological treatment at some point in their lifetime.

### Clinical outcomes

Machine learning was used to predict three treatment outcomes: the Hamilton Rating Scale for Depression (HRSD; Hamilton, 1960), Sheehan Disability Scale (SDS; Sheehan *et al*., 1996), and the Well-Being subscale of the Inventory of Depressive Symptoms (IDAS; Watson *et al*., 2008). The SDS and IDAS Well-Being were selected because they are important correlates of depression not well captured by the HRSD, were measured at the same time as the HRSD (i.e. at pre- and post-treatment), and, like the HRSD, showed substantial individual differences in treatment outcome. For more information about the psychometrics of the clinical outcomes, see online Supplementary materials, section 1.0.

### Candidate predictors

Given the exploratory, data-driven approach to analyses, all available data were utilized for each clinical post-treatment outcome to identify the most important candidate predictors. Total score and subscale scores if available (or individual items if not) were used as potential candidate predictors for the following self-report scales measured at pre-treatment: QIDS-SR, HRSD, Psychiatric Diagnostic Screening Questionnaire (PDSQ; Zimmerman and Mattia, 2001), SDS, IDAS Ill Temper, IDAS Well-Being, IDAS Social Anxiety, IDAS Traumatic Intrusion, IDAS Panic, Treatment Credibility and Expectancies Questionnaire (Devilly and Borkovec, 2000), and the Risky Families Questionnaire (Taylor *et al*., 2004). Clinical candidate predictors were also included, such as past and current psychotherapy, other health problems, family history of mental illness, antidepressant usage, and participant demographic variables, such as age, marital status, household size and composition, income, education level, sexual orientation, and ethnicity.

Given the geographic diversity of our participants, environmental context variables were also obtained by joining participant data to population databases (e.g. census data) based on participants’ zip code (postal address). Examples of available candidate predictors based on zip code of the participant included median household income, ethnic/racial diversity, crime rate, and access to mental healthcare providers. We also included the amount of time the participant spent on each Deprexis module to account for the potential impact of engagement with particular modules. Although these values are unknown prior to treatment, this aspect of the model could be useful when predicting outcomes for new patients by forecasting a range of expected outcomes that assume minimal *v.* maximal treatment engagement. For more detail on how these predictors were obtained, see online Supplementary materials, section 2.0. There were 120 candidate predictors in total used for each machine learning model. For a list of all predictors, see online Supplementary materials, section 3.0.

### Learning algorithms and tuning parameters

We selected two popular machine learning algorithms – the elastic net and the random forest – that perform well with samples of this size (<300). While large for a typical psychosocial intervention study, this would be considered small for many machine learning algorithms. The reason is that most machine learners (e.g. gradient boosting machines) require ‘tuning’ of several hyperparameters to achieve good predictive performance, which is typically done by trying out hundreds of different combinations of hyperparameter values by fitting hundreds of models and selecting the one that achieves the best cross-validation performance. This can result in overfitting, not due to an overly complex model, but rather having tested too many models (Ng, 1997). As noted by other researchers (Varma and Simon, 2006; Cawley and Talbot, 2010), overfitting from model selection will be most severe when the sample of data is small and the number of hyperparameters is large. For small data sets, Cawley and Talbot (2010) recommend avoiding hyperparameter tuning altogether by using an ensemble approach, such as random forests, that performs well without tuning.

While random forests have a few hyperparameters that could be tuned in principle, the default values for these typically work very well in practice: (1) the number of trees was set to 500. As shown by Breiman (2001), random forests do not overfit as more trees are added, but rather the generalization error converges to a limiting value. Thus, for this parameter, we only needed to verify that the default value was sufficient for the error to reach its plateau (James *et al*., 2013). For more detail, see online Supplementary materials, section 4.0. (2) The depth to which trees are grown was set to terminate if the additional split resulted in fewer than five observations in the terminal node. While using fully grown trees can overfit the data, Hastie *et al.* (2009) recommend not tuning this parameter because it seldom makes a substantive difference. (3) The number of variables that are randomly sampled when determining a split was set to the recommended value for regression problems, which is 1/3 of the candidate predictors. Tuning this parameter can make a substantive difference in performance, conditional on the prevalence of relevant *v.* irrelevant variables: this value should be large enough that any subset of variables of this size contains at least one relevant predictor.

The elastic net, on the other hand, has one parameter, *λ*, the magnitude of the shrinkage penalty that must be tuned with cross-validation, and an additional parameter, *α*, the proportion of L1 ‘lasso’ penalty (sum of absolute values of all coefficients) *v.* L2 ‘ridge’ penalty (sum of squared values of all coefficients), that often benefits from a small amount of tuning. For each outcome, we searched over 100 possible values of *λ* (autogenerated by the model fitting program) and three possible values of *α*: 0.01 (favoring the inclusion of most variables), 0.99 (favoring sparsity), and 0.5 (an equal mix of both L1 and L2). For reasons already discussed, cross-validation errors used for model selection are prone to optimistic bias. In the case of the elastic net, the models’ linear constraints might be expected to offset any optimistic bias introduced by this minimal amount of tuning. Nonetheless, to obtain an unbiased estimate of test error, we used a nested cross-validation procedure (Varma and Simon, 2006), which will be explained further in the *Prediction metrics* section. For the HRSD outcome, the value of *α* that minimized cross-validation error tended to be either 0.01 or 0.5 (mean = 0.31), with optimal *λ* ranging from 3.9 to 7.6 (mean = 5.7). Sparser models were selected for the SDS and IDAS outcomes, with optimal *α* tending to be 0.5 but sometimes 0.99 (mean = 0.66 for SDS and 0.63 for IDAS well-being). Shrinkage penalties were stronger for SDS (mean *λ* = 3.7) than IDAS well-being (mean *λ* = 0.73), indicating there were fewer relevant predictors of SDS.

### Comparing elastic net and random forest

The major appeal of the elastic net is that it offers a regularized version of the familiar linear model, whereas the major appeal of the random forest is that it offers an automated method of detecting non-linear and interaction effects. Importantly, both techniques allow all variables to ‘have their say’ (Hastie *et al.*, 2009, p. 601), i.e. they are able to accommodate multiple weak influences and weed out useless predictors, albeit through different mechanisms. The elastic net does this by directly fitting all predictors to the outcome simultaneously, while an L2 penalty shrinks the coefficients of useful but redundant predictors toward 0 and each other, and an L1 penalty shrinks the coefficients of useless predictors all the way to 0 (Zou and Hastie, 2005). The random forest achieves a similar end result because each regression tree in the forest ‘sees’ a different one-third of the predictors every time it chooses the best variable with which to recursively partition the data. Thus, the strongest predictors will not always be available, giving weaker predictors a chance to contribute. Since the predictions of the individual trees are averaged to yield an ensemble prediction, the contribution of any one predictor is effectively reduced. Other advantages of the random forest over the elastic net include less sensitivity to the effects of skewed distributions and outliers and an ability to work with the predictors in their natural units; for the elastic net to work, all variables, including dummy codes for factors, must be scaled to have the same mean and variance. On the other hand, if there are true linear relationships, the elastic net will be better at modeling them; the random forest can only approximate linear relationships as an average of step functions.

Our initial plan was simply to evaluate how each of these approaches performed on the Deprexis data individually. However, we later reasoned that averaging the predictions of the random forest with those of the elastic net (which are highly correlated – see online Supplementary materials, section 6.0–6.2) might retain the ‘best of both worlds’ and result in a superior prediction than either approach alone. This is not a novel concept; there are many examples of ‘blending’ or ‘stacking’ machine learners, and this is the basis of the so-called ‘super learner’ algorithm (van der Laan *et al*., 2007).

In this approach, a meta-learner is trained to optimize how models are combined, essentially resulting in a weighted average of model predictions. We would have liked to have used this approach here, but this would have required additional hold-out data or an independent test sample to obtain accurate estimates of model generalization, which our modest sample size could not support. Therefore, we adopted the simpler *committee method* (Hastie *et al.*, 2009, p. 289) of taking the unweighted average of the predictions from each model. Notably, the random forest itself is an ensemble of very complicated trees (each of which individually overfits the data such that its predictions will not generalize well to the population as a whole), and it already uses the committee method to average these unbiased but variable predictions into a stable, generalizable prediction. Essentially, we are simply adding the elastic net to the random forest ensemble – the final ensemble includes 500 regression trees + 1 elastic net – but with the elastic net's predictions weighted 500 times greater than those of an individual regression tree.

### Benchmark models

There is a growing recognition that many prior machine learning studies predicting health-related outcomes have compared machine learning models to relatively weak baseline models, such as the null or no information model (DeMasi *et al*., 2017). This is a very low threshold for the machine learning models to improve upon, thus producing falsely optimistic conclusions. Rather than a null model, the current study examined whether machine learning ensembles explained additional variance beyond a linear regression ‘benchmark’ model that predicted the clinical outcome with the same assessment at pre-treatment. Thus, for each outcome, we first report variance explained by the benchmark model and then examine whether the ensemble predicts variance not already explained by the benchmark model (i.e. model gain).

### Prediction metrics and cross-validation

An important aspect of model performance is how well it performs on cases that it was not trained on. We used 10-fold cross-validation to estimate a *predictive R*^2^ 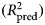, which is the fraction of variance in previously unseen data that the model is expected to explain. The 10-fold cross-validation was repeated 10 times using different randomized partitions of the data.^1^^†^ Within each repetition, 10 models were fit, each trained to 90% of the data and used to predict the outcomes for the 10% of cases that were withheld. An 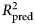 was then calculated based on the residual errors of the holdout predictions and then averaged across the 10 repetitions. This procedure was applied to both the benchmark model (see above) and the ensemble model. For the elastic net model, an additional 10-fold cross-validation procedure was nested within each 90% partition of data used for training and used to select values for the tuning parameters as described in the above section *Learning algorithms and tuning parameters*. The mean 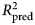 of the benchmark model was subtracted from the mean 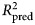 of the ensemble model to yield an estimate of *predictive gain* (Δ*R*^2^_pred_). A 95% CI for predictive gain was estimated as the 0.025 and 0.975 quantiles of the distribution of Δ*R*^2^_pred_ recomputed over 10 000 bootstrap replicates. For the elastic net, these numbers reflect the outer cross-validation, not the nested cross-validation, which tended to show a 1% (range = 0–3%) greater 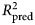, indicating that the estimates of error from the tuning procedure incur a slight optimistic bias.

Cross-validation of models was expedited by fitting models to resampled data in parallel on the Wrangler computing cluster provided by the Texas Advanced Computing Center (TACC) at the University of Texas at Austin (https://www.tacc.utexas.edu/). All analyses were implemented in R (version 3.4). Our code made extensive use of the *tidyverse* (Wickham, 2018) packages *dplyr*, *purrr*, and *tidyr* for general data extraction and transformation. Figures were generated using the packages *ggplot2* (Wickham, 2009), *gridExtra* (Auguie, 2017), and *ggmap* (Kahle and Wickham, 2013). The *randomForest* (Liaw and Wiener, 2002) and *glmnet* (Friedman *et al*., 2010) packages were used to implement the machine learning ensembles.

### Calculation of variable importance scores

We provide a variable importance metric that was obtained by selecting a sequence of values between the minimum and maximum of each predictor while holding all other predictors constant (at the mean of numeric variables or at the mode of factors), using the final model to make predictions for each of these selected values, and then obtaining the difference between the minimum and maximum prediction. This corresponds to the amount of partial influence that each variable had on the model prediction, in terms of the difference in outcome units that it was able to effect on its own. These predictions are for the final ensemble, thus the prediction for each variable is an average of linear and non-linear associations (from the elastic net and random forests models, respectively) between the predictor variable and outcome. Partial influence for each important predictor is illustrated in the partial dependence plots presented in the Results section.

In the online Supplementary material (sections 6.0–6.2), we also provide variable importance for the elastic net and random forest models separately. For the elastic net, variable importance was quantified as the absolute value of the standardized regression coefficient for each predictor. For the random forest, predictor importance was quantified as the percent mean increase in the residual squared prediction error on the out-of-bag samples^2^ when a given variable was randomly permuted. In other words, if permuting a variable substantially worsens the quality of the prediction, then it is important; if permutation has no impact on the prediction, then it is not important. We provide both sets of importance metrics because variable importance is not a well-defined concept (Grömping, 2009) and there are a large number of ways of computing variable importance (Wei *et al*., 2015). To facilitate comparison between the two metrics, both were scaled so that the importance scores of all variables sum to 1. For the most part, the importance metrics identify similar sets of predictors as important.

### Missing data

Imputation of missing predictor data was performed using the missForest method (Stekhoven and Bühlmann, 2012), which regresses each variable against all other variables and predicts missing data using random forests. Several studies have demonstrated the superiority of missForest over alternative approaches, such as *k*-nearest neighbors, multivariate imputation using chained equation, and several other random forest missing data algorithms (van Buuren, 2007; Waljee *et al*., 2013; Shah *et al*., 2014). Imputation was only applied to the missing predictors; none of the outcome data were used in the imputation, so there was no opportunity for information about the outcome to ‘leak’ into the predictor values and thus artificially improve prediction.

## Results

### HRSD

**Fig. 2.**
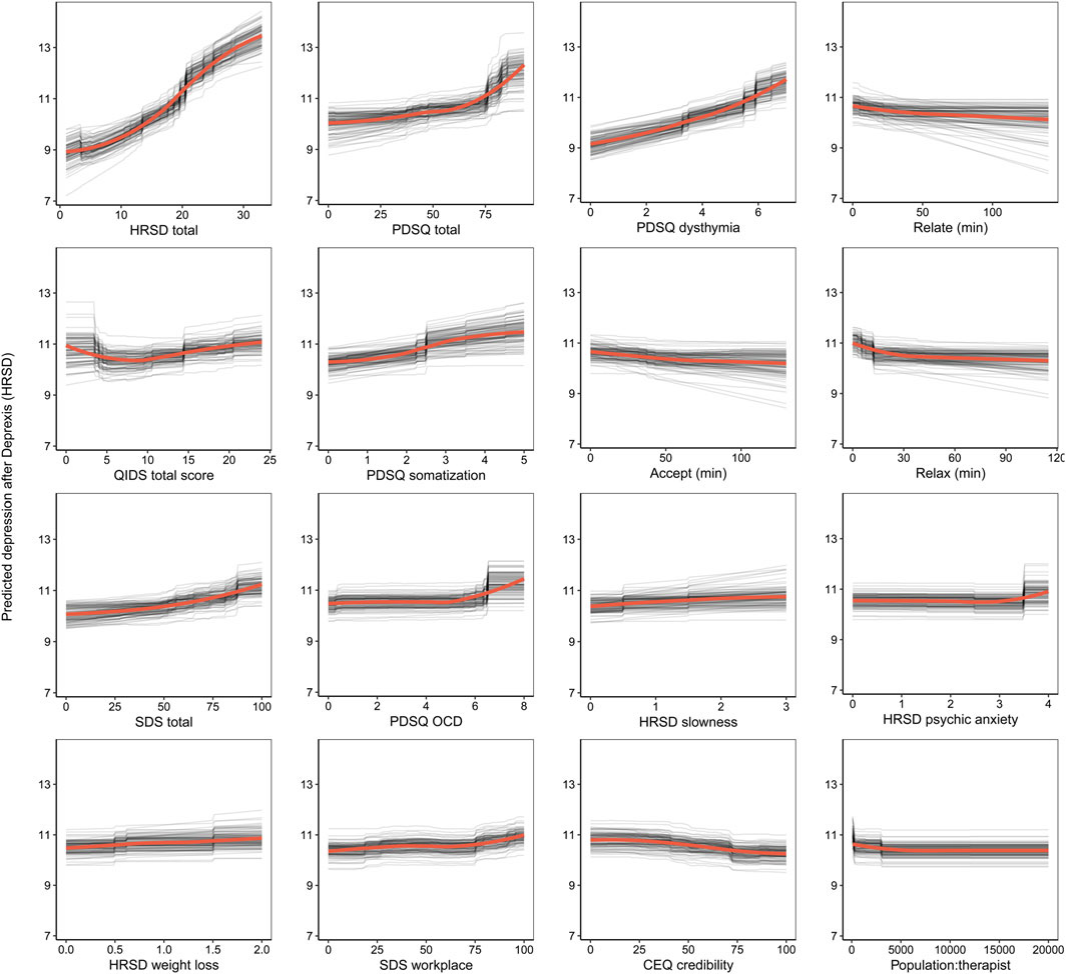
Partial dependence plots for the top 16 predictors of post-treatment interviewer-rated depression symptoms.

The benchmark model predicted 16.6% of the variance in post-treatment HRSD. The ensemble model predicted an additional 8% of the variance in post-treatment HRSD (see Table 1). The most important predictor variables were pre-treatment depression total score, psychiatric comorbidity, dysthymia, several depression symptom items, usage of several Deprexis modules, disability, treatment credibility, and availability of therapists (see Fig. 2).

**Table 1.** Prediction of post-treatment depression by linear regression model including only pre-treatment assessment of outcome (benchmark), additional variance explained beyond benchmark model by ensemble model (model gain), and total variance explained.

	Prediction *R*^2^	95% CI
HRSD		
Benchmark	0.17	0.07–0.26
Random forest	0.23	0.14–0.31
Elastic net	0.24	0.14–0.33
Random forest/elastic net ensemble	0.25	0.16–0.33
**Gain for ensemble model**	**+0.08**	**+0.008 to +0.15**
Disability		
Benchmark	0.20	0.10–0.31
Random forest	0.24	0.13–0.34
Elastic net	0.24	0.15–0.33
Random forest/elastic net ensemble	0.25	0.16–0.35
**Gain for ensemble model**	**+0.05**	**−0.003 to +0.10**
IDAS-Well Being		
Benchmark	0.18	0.08–0.27
Random forest	0.26	0.19–0.34
Elastic net	0.29	0.19–0.40
Random forest/elastic net ensemble	0.29	0.21–0.38
**Gain for ensemble model**	**+0.12**	**+0.05 to +0.19**

95% CIs for prediction *R*^2^ were based on the standard error formula applied to the 10 × 10 cross-validation estimates; 95% CIs for gain (the increase in predicted *R*^2^ over benchmark) were estimated by bootstrap.

Not surprisingly, partial dependence plots indicated a fairly linear relationship for pre-treatment HRSD and dysthymia; as pre-treatment depression/dysthymia increased, so did predicted post-treatment HRSD. Psychiatric comorbidity had a more curvilinear relationship, as post-treatment HRSD gently increased with increasing comorbidity until relatively high levels of comorbidity where post-treatment HRSD increased much more quickly. Higher levels of specific symptoms of depression, including slowness, psychic anxiety, and weight loss, were associated with higher post-treatment depression; disability related to psychiatric symptoms had a similar association.

Notably, usage of the relationships, acceptance, and relaxation modules were identified as important predictors. Using these modules for at least 30 min was associated with a 1.2-point greater reduction in HRSD score (all other predictors being equal), which is approximately one-quarter of the mean outcome difference observed for Deprexis-treated *v.* wait-list groups. As can be seen in many of the partial dependence plots for the 16 highest impact predictors (Fig. 2), associations between predictors and post-treatment HRSD were often non-linear and effects were relatively small (with the exception of the first three variables).^3^ Importance scores are also presented separately for the random forests and elastic net models in online Supplementary materials, section 6.0.

### Symptom-related disability

Consistent with prior work, to create a disability outcome, the work, social, and family disability questions (three items in total) from the SDS were averaged to form a single index of symptom-related disability. The benchmark model with pre-treatment disability predicted 20.4% of the variance in post-treatment symptom-related disability. The ensemble model predicted an additional 5% of the variance in post-treatment disability (Table 1).

**Fig. 3.**
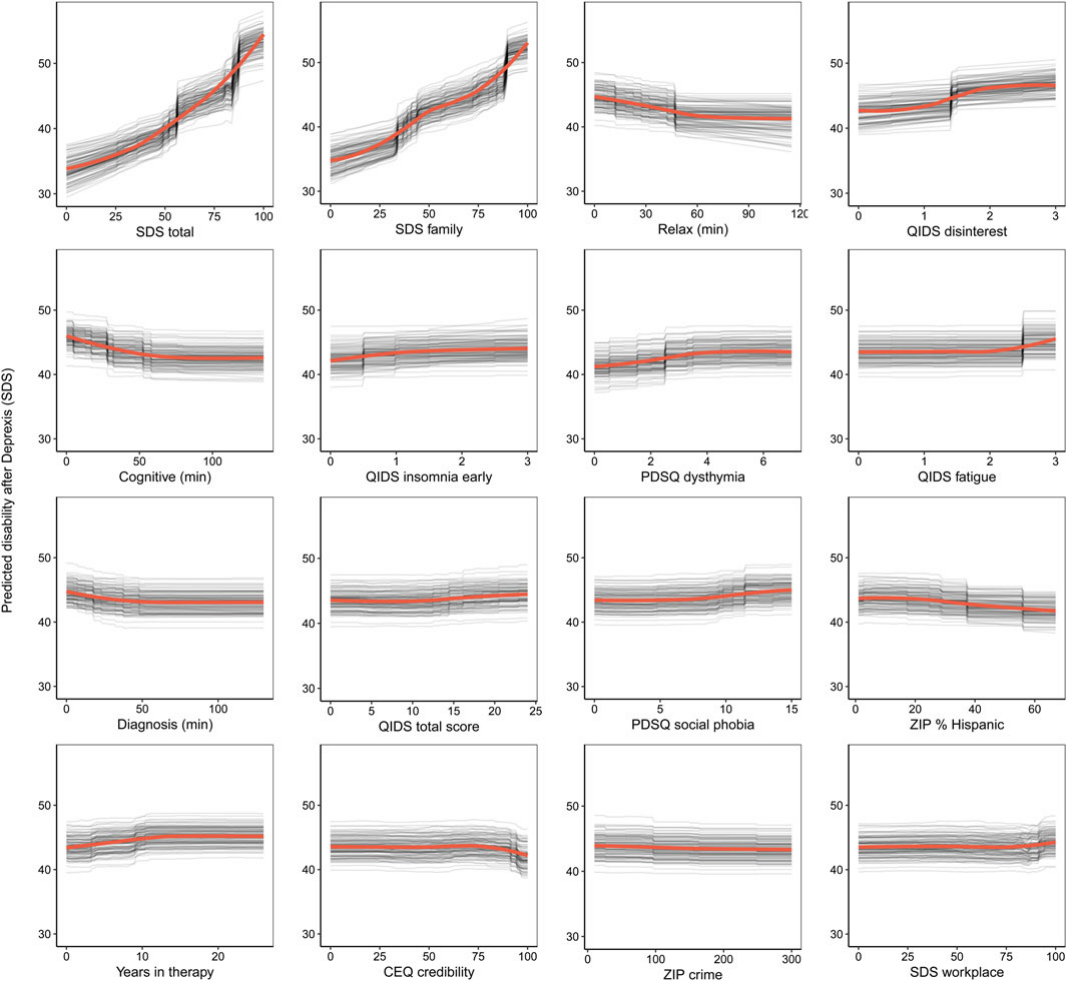
Partial dependence plots for the top 16 predictors of post-treatment disability.

As can be seen in Fig. 3, the pre-treatment disability composite had the strongest importance score, which was approximately equivalent to the importance of disability in the family domain. Nevertheless, several other variables also contributed to the prediction of disability. Several QIDS-SR items were identified as important predictors, including disinterest, early insomnia, and fatigue. More time spent on both the relaxation and cognitive modules (the benefit tapered off after approximately 60 min on each module) were both associated with lower post-treatment disability. Higher percentage of zip code with Hispanic ethnicity and fewer years in therapy were also associated with lower disability. Importance scores are also presented separately for the random forests and elastic net models in online Supplementary materials, section 6.1.

### Depression-related well-being

**Fig. 4.**
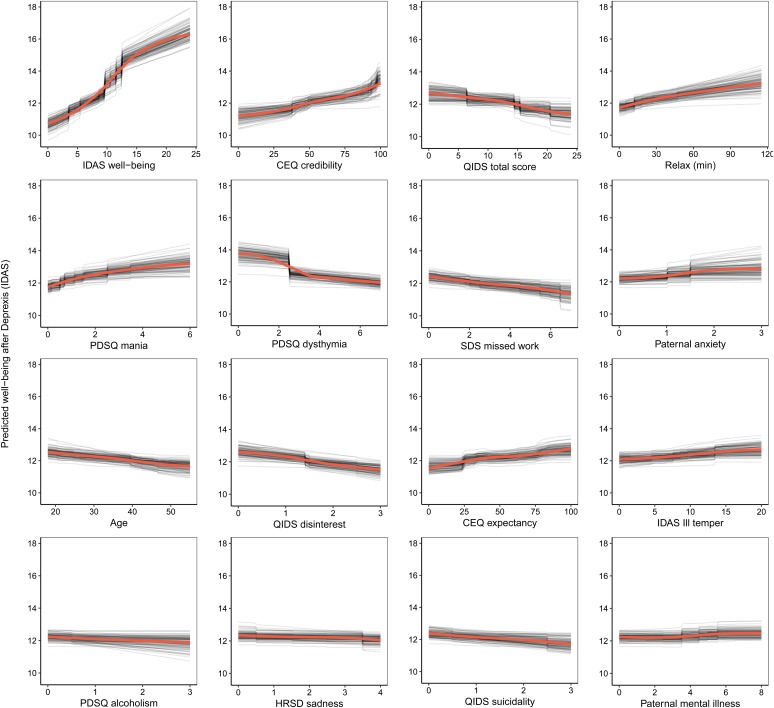
Partial dependence plots for the top 16 predictors of post-treatment well-being (low positive affect) symptoms.

The benchmark model with pre-treatment well-being (positive affect) predicted 17.8% of the variance in post-treatment symptom-related well-being. The ensemble model explained an additional 11.6% of the variance in post-treatment well-being (Table 1). As can be seen in Fig. 4, not surprisingly, higher pre-treatment well-being was associated with higher post-treatment well-being.

Comorbid psychopathology was also an important predictor of post-treatment well-being. The most important forms of comorbidity included mania symptoms, dysthymia, disinterest, and ill temper. Higher perceived treatment credibility and greater confidence that treatment would help were both associated with greater improvements in well-being in a fairly linear fashion (see Fig. 4). Younger age was associated with a better outcome as was paternal anxiety and mental illness. Higher use of the relaxation module was also associated with better post-treatment well-being. Importance scores are also presented separately for the random forests and elastic net models in online Supplementary materials, section 6.2.

### Deprexis module usage

**Fig. 5.**
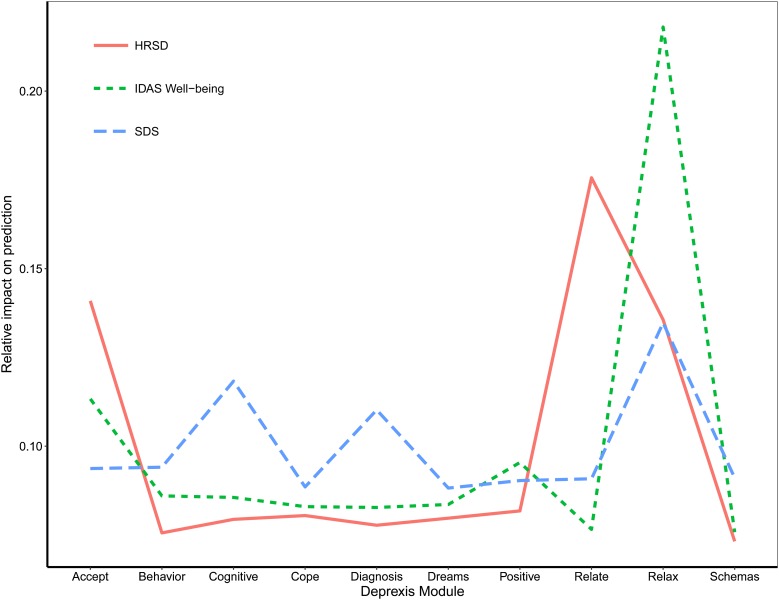
Importance of Deprexis module usage for predicting post-treatment depression, disability, and well-being (positive affect).

A final plot highlights the relative impact of module usage for each of the three outcomes (see Fig. 5). To generate these scores, the module importance scores for each outcome were scaled to sum to 1. A few notable patterns emerge. First, the most important module appeared to be the relaxation module; greater usage was associated with fewer depression symptoms, less disability, and more well-being. In addition, usage of the acceptance and relationship modules were most important for the prediction of HRSD depression symptoms. The cognitive module was important for predicting reductions in disability, as was the diagnosis module. Time spent on most of the other modules was not strongly associated with symptom improvement, at least for the average user. It is important to note that usage of all modules did factor (weakly) into the final prediction, and modules that were not important for the average user might nonetheless be very important when predicting the outcomes for some individuals.

## Discussion

This study used an ensemble of random forests regression trees and elastic net machine learning approaches to predict symptom change in response to Deprexis, an Internet intervention for depression. In contrast to many prior studies (DeMasi *et al*., 2017), we determined whether the ensemble predicted significantly more variance in post-treatment outcomes than a benchmark linear regression model that only included the baseline assessment of the outcome. Candidate predictors ranged from patient demographics, to comorbid psychopathology, past and current treatment utilization, to environmental characteristics based on the participants’ residence (i.e. postal code in the USA). In summary, the ensemble outperformed an autoregressive linear benchmark model for the prediction of interviewer-rated depression symptoms (8% gain in variance explained, 25% total variance explained), symptom-related disability (5% gain in variance explained, 25% total variance explained), and well-being (12% gain in variance explained, 29% total variance explained).

Results provide important new insight into who is most likely to respond to Internet interventions such as Deprexis. Not unexpectedly, pre-treatment assessments were the strongest predictors of each outcome. However, a number of additional predictors, all making relatively small contributions to the prediction, were associated with symptomatic improvement. The ensemble indicated that comorbid psychopathology was an especially important predictor of post-treatment HRSD, particularly total psychopathology and dysthymia (other psychopathology symptoms were also identified as important predictors). This may not be surprising, as past research has indicated that comorbid psychopathology is often associated with poor treatment response (Smits *et al*., 2012). Also consistent with prior research, pre-treatment disability was an important predictor of symptom change (Kessler *et al*., 2017). High pre-treatment impairment has been shown to predict poor treatment response across both pharmacological and psychological treatment modalities (Trivedi *et al*., 2006; Frank *et al*., 2011; Jarrett *et al*., 2013), but does not appear to moderate response to any specific form of treatment (Kessler *et al*., 2017). Nevertheless, impairment appears to be an important, non-specific predictor of treatment response.

The ensemble was also able to predict additional variance in post-treatment well-being beyond the benchmark model. Comorbid dysthymia, disinterest, depression symptoms, and mania symptoms were all important predictors of well-being. Further, treatment credibility and treatment expectancies were relatively strong predictors of symptom change. Self-guided interventions should consider incorporating interventions that enhance patient expectations about treatment effectiveness and treatment credibility, as initial expectancies have predicted treatment engagement and outcome in psychotherapy and Internet treatments (Greenberg *et al*., 2006). Although positive expectations are sometimes dismissed as mere ‘placebo’ processes, which need to be controlled in trials, we concur with others that such factors can fuel treatment engagement and improve outcomes (Kirsch *et al*., 2016). As with the HRSD, disability across multiple domains was also an important predictor of post-treatment well-being.

A final aim was to examine the differential association of Deprexis module usage to the three post-treatment outcomes. Usage of the relationships, acceptance, and relaxation modules appeared to be the most closely associated with the depression-related outcomes. Notably, this study is among the first to identify specific module dosages that are associated with symptom improvement. For each of the modules, symptom reduction appeared to taper off after approximately 30–50 min of usage. Prior work has found that module completion is modestly associated with improvements in depression (Spek *et al*., 2007). Much of this prior work has examined a linear relationship between usage and symptom improvement, even though there is evidence that this association may be non-linear (Donkin *et al*., 2013). One benefit of using random forests together with elastic nets is that random forests can detect non-linear associations without having to specify the form of the association in advance. The use of standardized interventions (as opposed to face-to-face interventions that can have variable implementations) also facilitates these dose–response analyses.

There are several limitations of this study that should be noted. First, future work predicting treatment response would benefit from using larger samples, which would allow for more complex models without overfitting. Although it would be ideal for future work to also include highly dimensional data, such as neuroimaging, hormones, genetics, and microbiota, it would not be desirable to include those data at the cost of large sample size and population diversity. In addition, without comparable data from an alternative clinical intervention, we have no way of gauging the extent to which this model is predicting response to Deprexis specifically *v.* a response to interventions more generally. We do not yet know whether the results of this model can be used to recommend whether an individual should try Deprexis or not. Finally, although Deprexis has been studied in other countries, this was the first trial of the English version of Deprexis, and many of the important predictors here were either not collected or not applicable to prior European trials. Thus, testing our algorithm with other Deprexis-treated samples was not possible.

Given that the goal of this project was to develop an algorithm that could be used to predict response to a single treatment, one might wonder how this work could be used in real-world clinical settings. Imagine a clinical setting with a large waiting list (not an unusual circumstance in some settings, such as treatment centers at large medical centers or universities). Patient information could be obtained, via an online survey or other methods, and fed into the Deprexis treatment response algorithm. The algorithm may predict several of these new patients will show significant improvement in symptoms following Deprexis, whereas other individuals are predicted to show relatively little symptom change.

As a result of these analyses, Deprexis could be provided right away to the first set of individuals, perhaps allowing for the patients predicted not to respond to Deprexis to be scheduled sooner for more intensive, in-person treatment. Treatment centers would need to decide the threshold for an acceptable amount of predicted change in order to receive Deprexis, and this could potentially dynamically fluctuate depending on demand for services, therapist availability, and other constraints. In addition, while a patient's future usage of the various modules is obviously unknown prior to treatment, this aspect of the model could be used to explore different usage scenarios and recommend which modules are likely to yield the greatest return on an individual patient's time investment. To make this hypothetical application more concrete, we provide case examples of predicted response to treatment, using the machine learning algorithm developed in this study, for two hypothetical patients under two different usage scenarios in online Supplementary materials, section 7.0.

This exercise demonstrates how some patients could be identified as good candidates for this low-intensity, Internet-based treatment, whereas others may not be. Of course, because we have only one treatment, we do not know how these participants might respond to other treatments (or no treatment at all). Ideally, algorithms could be developed for predicting response to different treatments. These algorithms could then be used to determine the optimal treatment(s) for a patient with a given set of attributes. We believe methods used in the current study provide a small but important step toward developing these algorithms and, given the emphasis of machine learning methods for reducing overfitting and increasing generalization to new samples, this may be a better alternative than traditional statistical approaches typically used to detect treatment predictors (for a review, see Cohen and DeRubeis, 2018). With further refinement, ensemble machine learning methods may facilitate a more efficient mental health care system by helping clinicians optimize treatment delivery so that patients initially receive the treatment with the best likelihood of a positive response for that specific individual.

## Footnotes

† The notes appear after the main text.

## Notes

1 One may choose the number of folds to be anywhere from two (equivalent to splitting the data in half) to the number of observations (equivalent to ‘leave-one-out’ cross-validation). This choice incurs a bias-variance tradeoff. Fewer folds mean smaller sample sizes for fitting the models and therefore worse fits (greater bias) but more similar performance between different samples (less variance); more folds mean larger sample sizes and therefore better fits (less bias) but more inconsistent performance between different samples (greater variance). Empirically, 10-fold cross-validation seems to offer the best compromise between these two extremes. Specifically, the 10 × 10 cross-validation tests performed here follow the work and recommendations of Bouckaert (2003; 2004) for reliably comparing the performance of different learning algorithms.

2 The term ‘out of bag’ derives from the term ‘bagging’, which is a portmanteau of ‘bootstrap aggregating’. Because each split in each regression tree in the random forest is based on a bootstrap sample of the original data, there are always a number of samples (about 1/3 on average) that are randomly left out of each ‘bagged’ sample. Hence, for the random forest, aggregating the prediction errors for these ‘out-of-bag’ observations provides an additional valid estimate of how well the machine learner can predict new cases.

3 Note that the self-reported depression symptoms were also assessed with the QIDS-SR. Results for the QIDS-SR are presented in online Supplemental materials, section 5.0.

## Supplementary material

For supplementary material accompanying this paper visit https://doi.org/10.1017/S003329171800315X. click here to view supplementary material
