# Supplementary material for: A machine learning ensemble to predict treatment outcomes following an Internet intervention for depression
Source: Psychol Med. 2018 Nov 5;49(14):2330–41. doi: 10.1017/S003329171800315X (PMC6763538; doi:10.1017/S003329171800315X)
Supplement: Supplementary file 1 [file S003329171800315Xsup001.docx]

**Supplemental Materials**

**Section 1.0: Additional detail regarding clinical outcomes**

The HRSD is a semi-structured interview consisting of 17 items. Items have 3 to 5 possible rating options that increase in severity with total scores ranging from 0 to 50. The HRSD has demonstrated good reliability and validity(Trajković et al. 2011). For the current study, 25 HRSD telephone interviews were recorded and scored independently by a second trained rater. Average score intraclass correlation between the raters’ HRSD total score ratings was very high: ICC = 0.98, 95% CI [.96, .99], *F*(24, 25) = 60.7, *p* < .001. Participants showed a significant mean improvement in HRSD score of 5.8 points, 95% CI [5.0, 6.7], Cohen’s *d* = 0.82, 95% CI [0.65, 0.99].

The SDS measures functional impairment due to symptoms in three related domains, work/school, family, and social life. There is a single item that measures impairment in each domain on a 0 to 100 scale. Scores above 50 on any of the three items is thought to indicate functional impairment and we operationalized a total disability score as the average of the three items. There are two additional questions that measure time missed from work in the past week and days unproductive in the past week. Prior work has demonstrated strong psychometric properties of the SDS(Sheehan et al. 1996). Participants showed a significant mean improvement in SDS scores of 17 percentage points, 95% CI [14, 20], Cohen’s *d* = 0.67, 95% CI [0.50, 0.84].

The IDAS measures a variety of depression symptoms that are not commonly measured by other self-report depression inventories. For the current study, we used the 8-item Well-Being subscale where participants indicated the extent to which they had experienced symptoms of high energy and positive affect during the past two weeks on a 5-point scale ranging from not at all (0) to extremely (4). Total scores could range from 0 to 32. Prior work has shown that the IDAS Well-Being subscale has good psychometric properties in clinical samples and that well-being is strongly inversely correlated with a diagnosis of Major Depressive Disorder(Watson et al. 2008). Participants showed a significant mean improvement in well-being of 4.7 points, 95% CI [3.9, 5.4], Cohen’s *d* = 0.74, 95% CI [0.56, 0.91].

**Section 2.0: Methods for obtaining zip-code related information**

Location-specific data about participant’s surrounding environment was collected using a combination of automated census data-mining and web-scraping with Python. Preliminarily, a database listing all valid U.S. zip codes was used to determine the status of each participant’s zip code ensuring that the zip code both exists and is residential. For zip codes that were discovered to be PO Boxes, an in-house algorithm using tools from geopy and pyzipcode was used to automatically map flagged PO Box zip codes to ‘surrogate’ residential zip codes using surrounding zip codes within a given square mile radius. That square mile distance threshold was arbitrarily set at 20 square miles for those PO boxes with a land area <= 5 square miles (i.e. to account for the closer proximity of adjacent zip codes in urban areas) and at 40 square miles for those PO boxes with a land area > 5 square miles. This procedure in turn yielded lists of approximately 15 or so candidate zip codes for each PO Box in question—a list that was further reduced to only those that were residential. Vicenty distance was then calculated for each of these remaining candidate zip codes to determine the one residential zip code that was geographically closest to the PO Box zip code.

Next, an in-house zip code search engine was developed in Python using a combination of web scraping tools, including pyzipcode, uszipcode, urllib, geopy, BeautifulSoup, requests, and Pandas packages. This web-scraping tool iterated sequential zip code lookups through a combination of the US Census API and two commercial web-pages: [moving.com](http://moving.com/) and [psychologytoday.com](http://psychologytoday.com/).

Several basic zip-code measures were then extracted: total population counts, land area, population density, average annual income, longitude, and latitude. Moving.com was additionally scraped using urllib to corroborate the US Census Bureau data, while also adding additional measures such as racial distribution statistics, weather information, and average commute times. Lastly, [psychologytoday.com](http://psychologytoday.com/) was automatically scraped for each zip code to count the number of unique mental healthcare professionals within a 5-mile radius of each participant’s zip code as a surrogate measure for mental healthcare access.

**Section 3.0: List of all predictors entered into machine learning models**

| **Predictor description** | **Succinct predictor label** |
| --- | --- |
| Received treatment immediately | Immediate treatment |
| QIDS: Trouble falling asleep | QIDS insomnia early |
| QIDS: Trouble staying asleep | QIDS insomnia middle |
| QIDS: Waking up too early | QIDS insomnia late |
| QIDS: Sleeping too much | QIDS hypersomnia |
| QIDS: Feeling sad | QIDS sad |
| QIDS: Appetite loss | QIDS appetite loss |
| QIDS: Appetite gain | QIDS appetite gain |
| QIDS: Weight loss | QIDS weight loss |
| QIDS: Weight gain | QIDS weight gain |
| QIDS: Trouble concentrating or making decisions | QIDS indecision |
| QIDS: Negative view of self | QIDS self-view |
| QIDS: Thoughts of death or suicide | QIDS suicidality |
| QIDS: Loss of general interest | QIDS disinterest |
| QIDS: Loss of energy | QIDS fatigue |
| QIDS: Feeling slowed down | QIDS slowness |
| QIDS: Feeling restless | QIDS agitation |
| QIDS total | QIDS total score |
| Age in years | Age |
| Married or in domestic partnership | Married |
| Number of children | Num. child |
| Number of children living at home | Num. child at home |
| Number of adults living at home | Num. adult at home |
| Gender | Gender |
| LGBTQ | LGBTQ |
| Hispanic or Latino ethnicity | Ethnicity |
| Education | Education |
| Annual household income ($) | Income |
| Number of drugs taken that inhibit norepinephrine reuptake | Norepinephrine |
| Number of drugs taken that inhibit serotonin reuptake | Serotonin |
| Number of drugs taken that inhibit dopamine reuptake | Bupropion |
| Number of benzodiazepines taken | Benzodiazepine |
| Number of years spent in psychotherapy | Years in therapy |
| Paternal history of anxiety disorders | Paternal anxiety |
| Paternal history of mood disorders | Paternal MDD |
| Paternal history of substance abuse | Paternal drug abuse |
| Paternal history of any mental illness | Paternal mental illness |
| Maternal history of anxiety disorders | Maternal anxiety |
| Maternal history of mood disorders | Maternal MDD |
| Maternal history of any mental illness | Maternal mental illness |
| Personal history of endocrine disorder | Endocrine disorder |
| Personal history of general health problems | Health problems |
| Personal history of early life stress | Early life stress |
| Geospatial latitude | ZIP latitude |
| Geospatial longitude | ZIP longitude |
| Population : therapist ratio | Population:therapist |
| Median age of area residents | ZIP median age |
| % area residents who are married | ZIP % married |
| % area residents who are white | ZIP % white |
| % area residents who are Hispanic | ZIP % Hispanic |
| Population density of residential area | ZIP pop density |
| Population change of residential area since 1980 | ZIP pop change 1980 |
| Number of years average resident has lived in community | ZIP years residency |
| Air quality index for residential area | ZIP air quality index |
| Median disposable annual income ($) of area residents | ZIP disposable income |
| Median travel time to work for area residents | ZIP commute time |
| Crime risk for residential area | ZIP crime |
| Mean education level of area residents | ZIP education |
| Average low temperature in winter | ZIP winter low |
| Average high temperature in summer | ZIP summer high |
| Risk of severe weather impact | ZIP weather |
| Receiving additional psychotherapy | Current therapy |
| IDAS: Ill temper (% max) | IDAS Ill temper |
| IDAS: Panic (% max) | IDAS panic |
| IDAS: Social anxiety (% max) | IDAS social anxiety |
| IDAS: Trauma (% max) | IDAS trauma |
| IDAS: Well being (% max) | IDAS well-being |
| PDSQ: % MDD symptoms endorsed | PDSQ MDD |
| PDSQ: % dysthymia symptoms endorsed | PDSQ dysthymia |
| PDSQ: % PTSD symptoms endorsed | PDSQ PTSD |
| PDSQ: % eating disorder symptoms endorsed | PDSQ eating disorder |
| PDSQ: % OCD symptoms endorsed | PDSQ OCD |
| PDSQ: % panic symptoms endorsed | PDSQ panic |
| PDSQ: % mania symptoms endorsed | PDSQ mania |
| PDSQ: % psychosis symptoms endorsed | PDSQ psychosis |
| PDSQ: % agoraphobia symptoms endorsed | PDSQ agoraphobia |
| PDSQ: % social phobia symptoms endorsed | PDSQ social phobia |
| PDSQ: % alcoholism symptoms endorsed | PDSQ alcoholism |
| PDSQ: % GAD symptoms endorsed | PDSQ GAD |
| PDSQ: % somatization symptoms endorsed | PDSQ somatization |
| PDSQ: % hypochondria symptoms endorsed | PDSQ hypochondria |
| PDSQ: % total psychopathology symptoms endorsed | PDSQ total |
| SDS: % workplace disability | SDS workplace |
| SDS: % social life disability | SDS social |
| SDS: % family life disability | SDS family |
| SDS: Number of missed work days in past week | SDS missed work |
| SDS: Number of unproductive days in past week | SDS not productive |
| SDS: % total disability | SDS total |
| CEQ: % confidence in credibility of Deprexis | CEQ credibility |
| CEQ: % confidence that Deprexis will help | CEQ expectancy |
| CSSRS: suicidal ideation | CSSRS ideation |
| CSSRS: suicidal planning | CSSRS intensity |
| CSSRS: suicidal behavior | CSSRS behavior |
| HRSD: Depressed mood | HRSD sadness |
| HRSD: Feelings of guilt | HRSD guilt |
| HRSD: Suicidality | HRSD suicidality |
| HRSD: Trouble falling asleep | HRSD insomnia early |
| HRSD: Trouble staying asleep | HRSD insomnia middle |
| HRSD: Waking up too early | HRSD insomnia late |
| HRSD: Loss of general interest | HRSD disinterest |
| HRSD: Feeling slowed down | HRSD slowness |
| HRSD: Feeling restless or fidgety | HRSD agitation |
| HRSD: Feeling tense or irritable | HRSD psychic anxiety |
| HRSD: Somatic symptoms of anxiety | HRSD soma anxiety |
| HRSD: Loss of appetite | HRSD appetite loss |
| HRSD: Loss of energy | HRSD fatigue |
| HRSD: Loss of libido | HRSD libido |
| HRSD: Hypochondria | HRSD hypochondria |
| HRSD: Loss of weight | HRSD weight loss |
| HRSD Total | HRSD total |
| Usage of "Accept" module (min) | Accept (min) |
| Usage of "Behavior" module (min) | Behavior (min) |
| Usage of "Cognitive" module (min) | Cognitive (min) |
| Usage of "Cope" module (min) | Cope (min) |
| Usage of "Diagnosis" module (min) | Diagnosis (min) |
| Usage of "Dreams" module (min) | Dreams (min) |
| Usage of "Positive" module (min) | Positive (min) |
| Usage of "Relate" module (min) | Relate (min) |
| Usage of "Relax" module (min) | Relax (min) |
| Usage of "Schemas" module (min) | Schemas (min) |

**Section 4.0: Mean square error presented as a function of number of trees used in random forest model for HRSD**

For the random forest models, we examined the relationship between number of trees used and mean square error to ensure that addition reductions in error were not observed at high levels of trees. As can be seen in the figure below for the prediction of HRSD, mean square error appears to plateau for the out-of-bag sample at approximately 100 trees. However, to be conservative, we set 500 trees as a maximum in all of our random forest models. A very similar pattern was observed for all the other outcomes, suggesting that error rates would not have substantively declined further had we used an even larger number of regression trees.


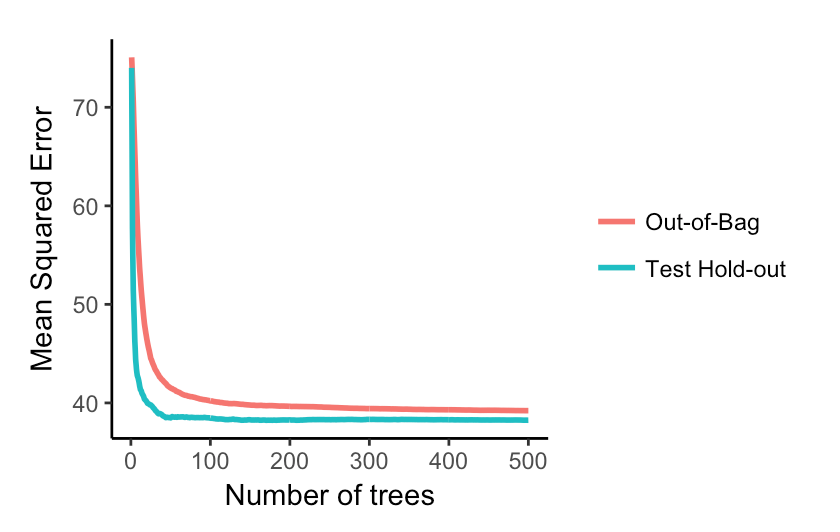


**Section 5.0: Prediction of post-treatment QIDS-SR.**

The QIDS-SR was obtained every two weeks during the clinical trial. However, to be consistent with the other analyses, only pre-treatment and post-treatment assessments are used in this analysis.

The benchmark model with pre-treatment QIDS-SR predicted 28.2% (range 27.4 – 28.7%) of the variance in post-treatment QIDS-SR. The blended ensemble explained an additional 2.7% (95% CI [-0.02, 0.08] of the variance in post-treatment QIDS-SR, which was not significantly different from zero. The most important predictors of post-treatment QIDS-SR score was pre-treatment QIDS-SR score followed by the QIDS-SR suicide item and total disability.

*Supplemental Figure 1: Partial dependence plots for the top sixteen predictors of post-treatment QIDS-SR*

As can be seen in the partial dependence plots, higher pre-treatment QIDS-SR score was associated with higher post-treatment QIDS-SR. The associations with suicidality and disability were more non-linear, with the association with post-treatment QIDS increasing towards the higher levels of suicidality and total disability. Other predictors, including years in therapy, fatigue, use of the relaxation module in Deprexis, and percentage of residents who were Hispanic all modestly contributed to the prediction. However, one should keep in mind that all predictors only explained 2.7% of the variance in post-treatment QIDS-SR beyond pre-treatment QIDS-SR.

**Section 6.0: HRSD supplemental analyses.**

There was strong agreement for the cross-validation hold-out predictions for post-treatment HRSD for the random forest and elastic net models.


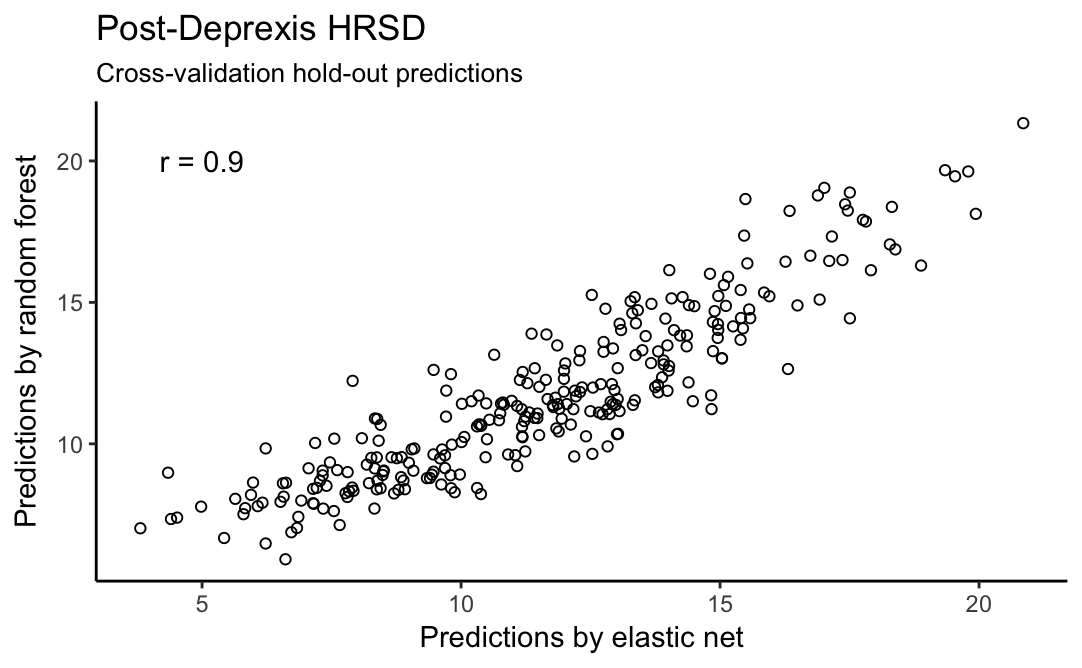


The 20 most important variables for the elastic net (A) and random forest (B) are presented below. Both models identified depression and dysthymia symptoms as most important. Psychiatric comorbidity also had high importance scores, with the RF identifying total comorbidity and elastic net identifying somatization as the most important forms of comorbidity. The remaining predictors had relatively modest importance scores. Converging predictors included disability, use of acceptance and relaxation modules, the depression symptom of weight loss, and treatment credibility.


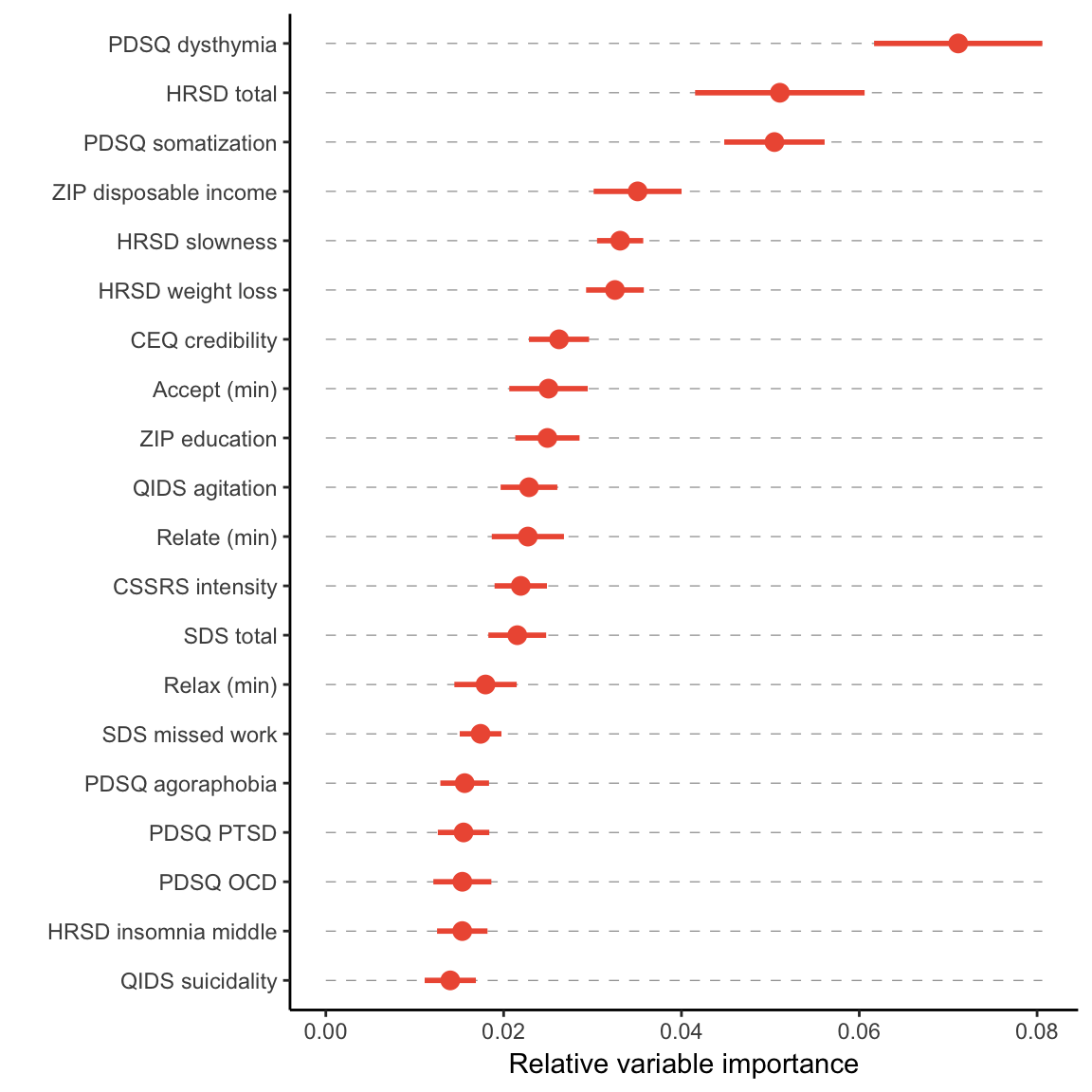


A

B


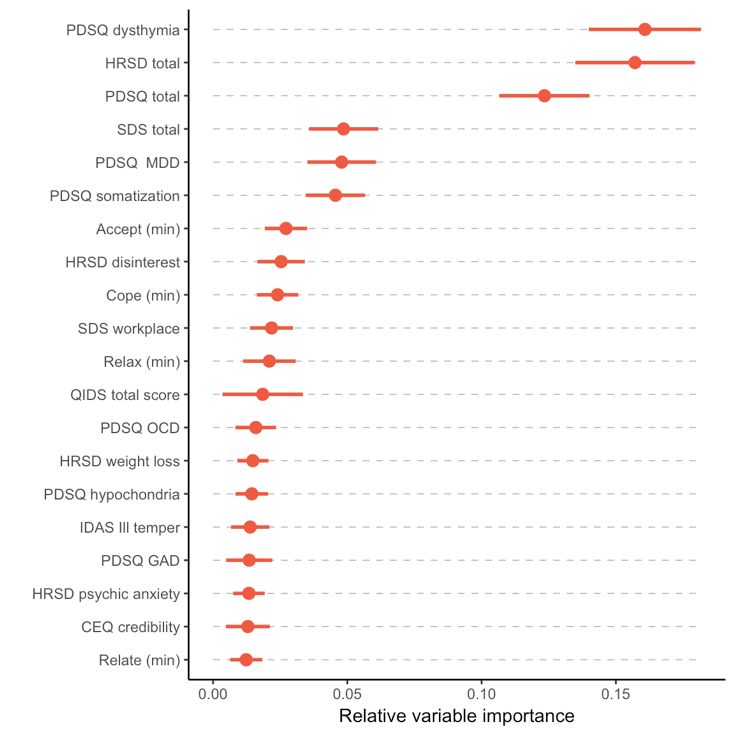


**Section 6.1: SDS supplemental analyses.**

There was strong agreement for the cross-validation hold-out predictions for post-treatment disability measured by the SDS for the random forest and elastic net models.


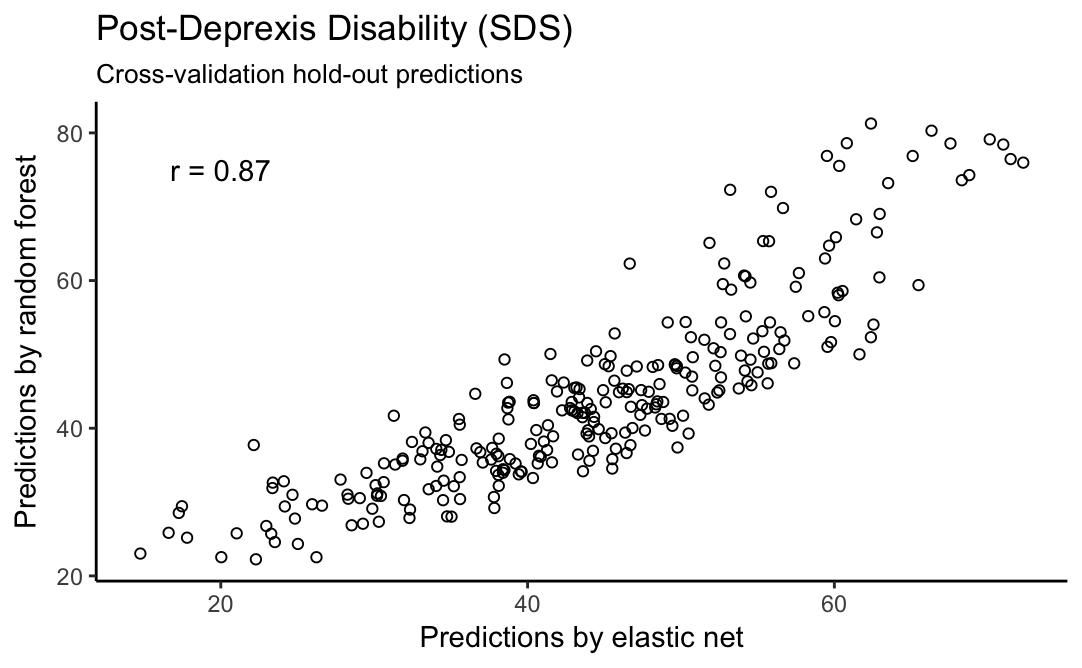


The 20 most important variables for the elastic net (A) and random forest (B) are presented below. Both models identified total disability and family-related disability to be highly important. The RF model also indicated that social disability was important. Depression symptoms also contributed to the prediction, with the elastic net identifying disinterest and insomnia as important, whereas the RF identified disinterest and fatigue.

B

A


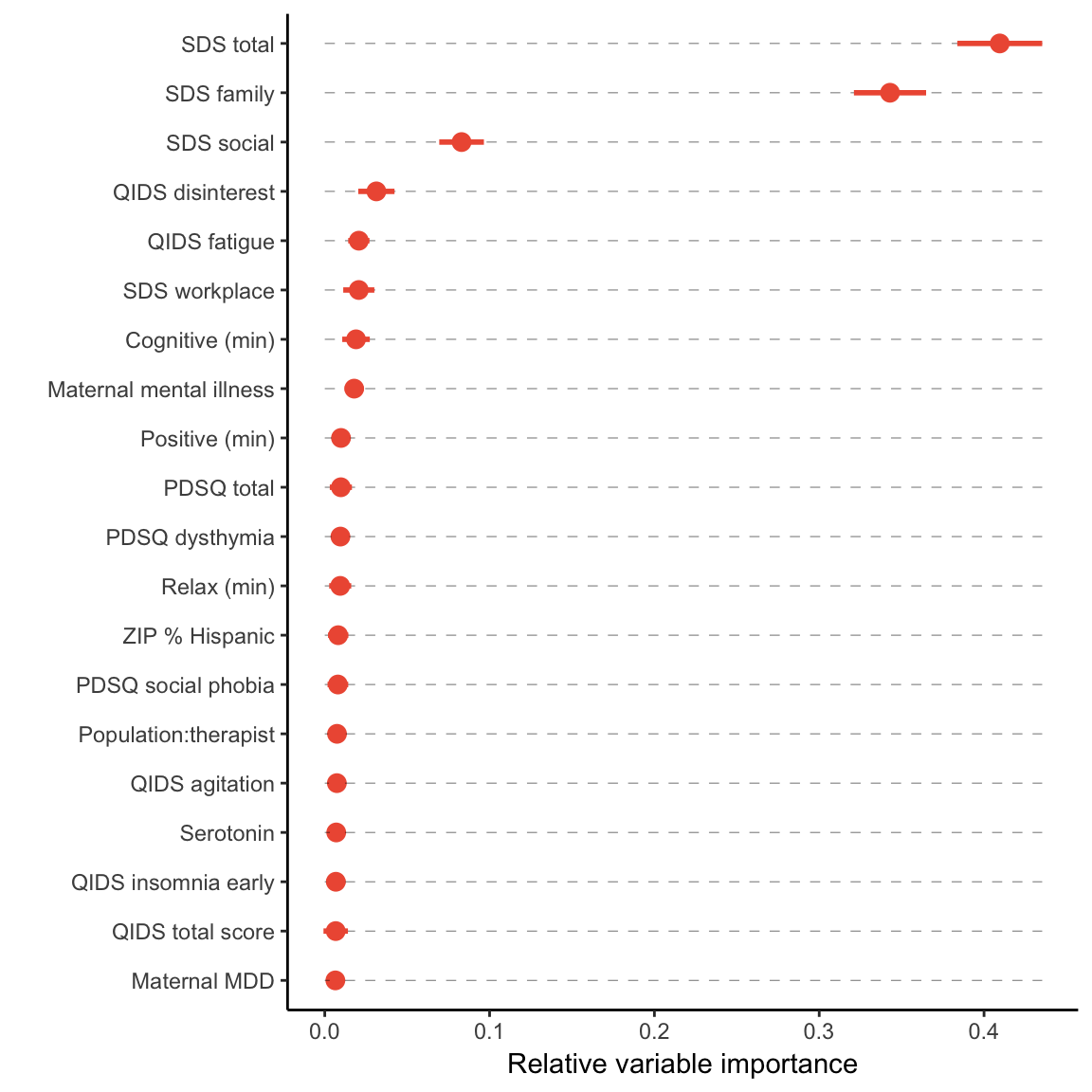

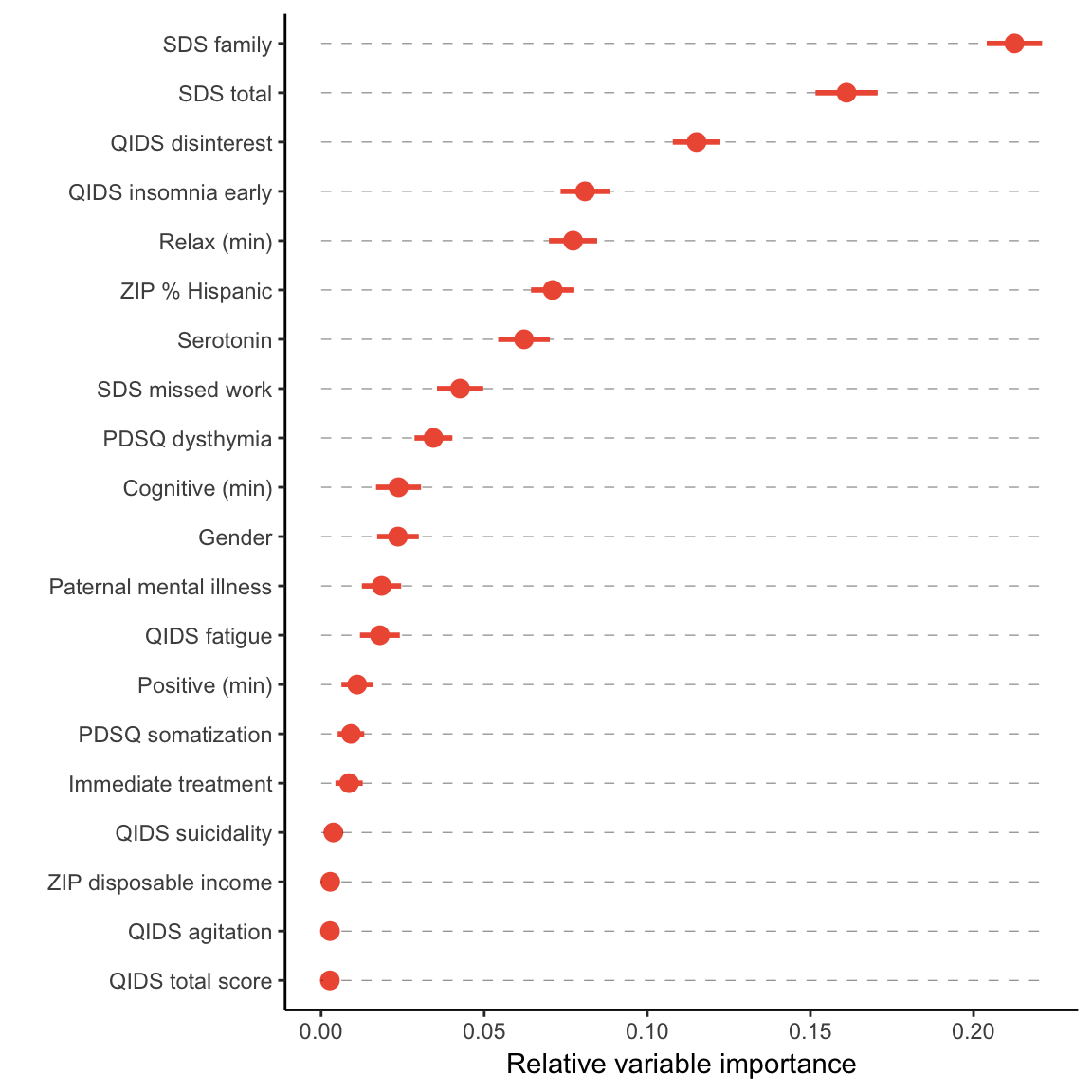


**Section 6.2: IDAS Well-Being supplemental analyses.**

There was strong agreement for the cross-validation hold-out predictions for post-treatment IDAS Well-Being for the random forest and elastic net models.


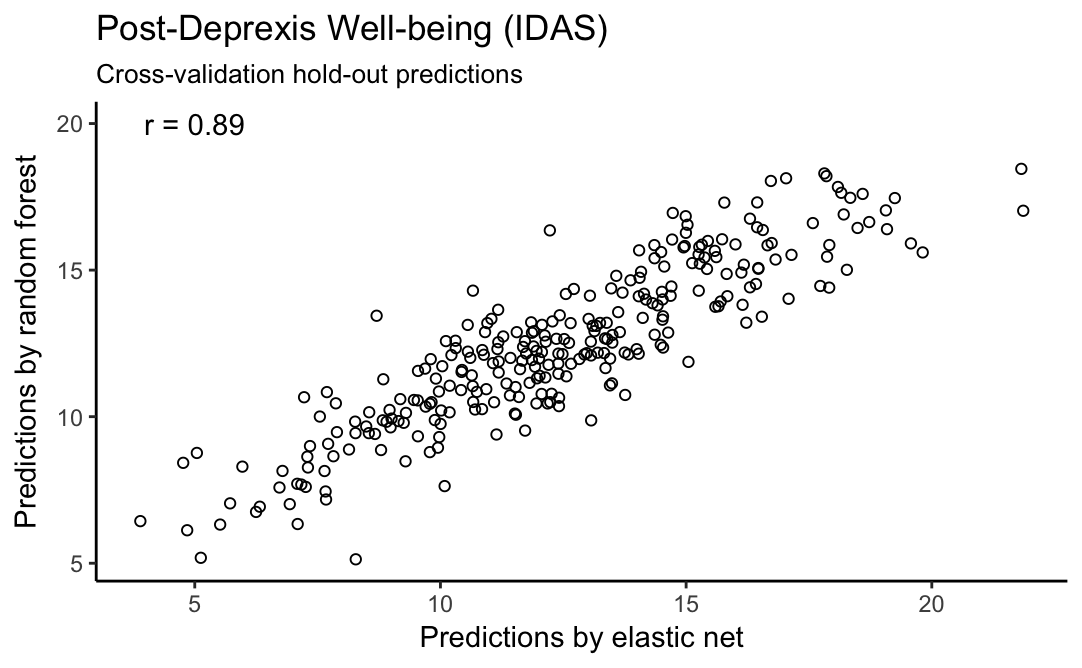


The 20 most important variables for the elastic net (A) and random forest (B) are presented below. For well-being, both models identified pre-treatment wellbeing as the strongest predictor of post-treatment well-being. The RF model identified several depression symptoms as important, including disinterest, QIDS-SR total score, dysthymia, mania, and treatment credibility. The elastic net identified the symptoms of disinterest, dysthymia, mania, and ill temper, as important predictors. Treatment credibility was also identified as important, in addition to age and use of the relaxation module.

A

B


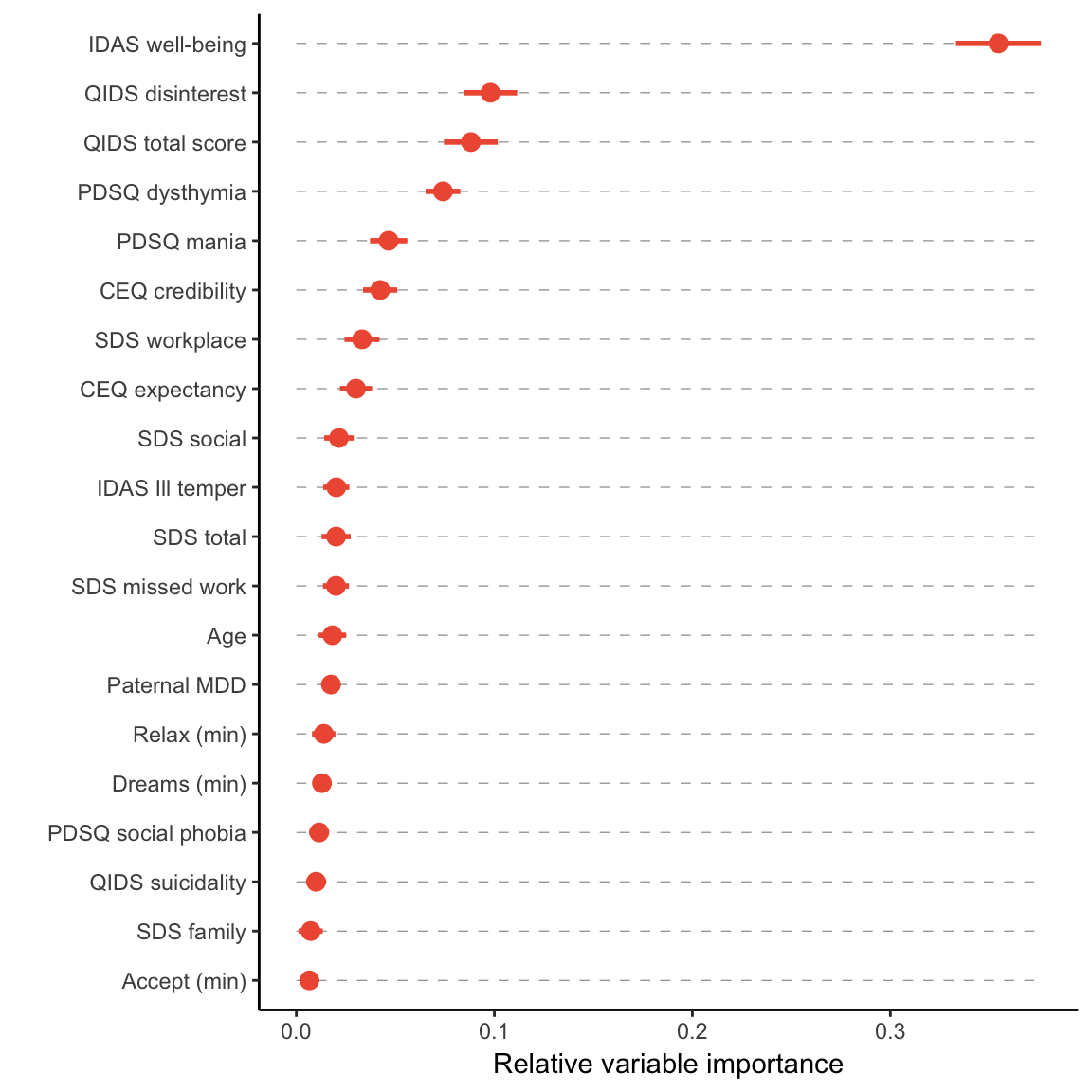

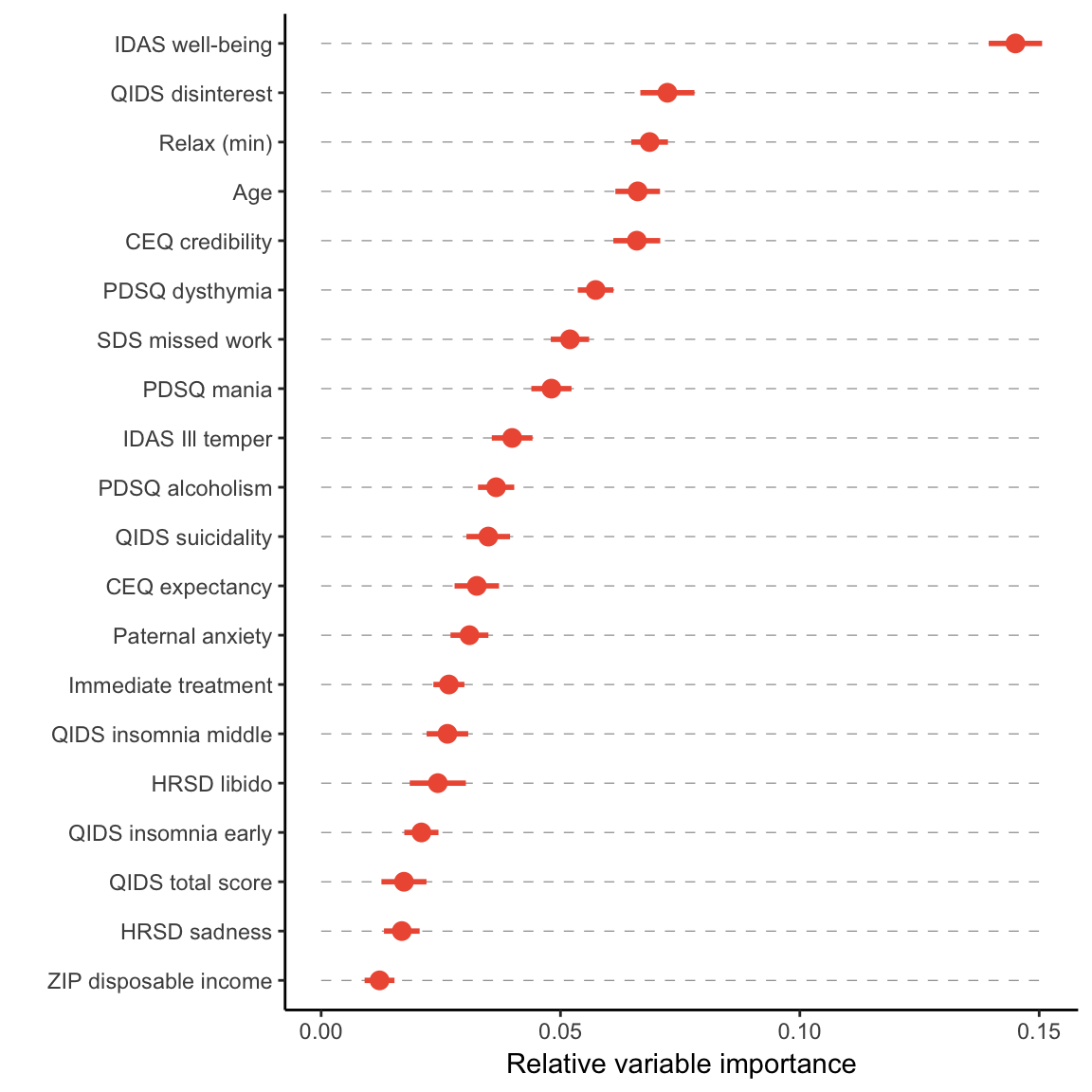


**Section 7.0: Actionable model prognoses for some hypothetical cases**

Here we provide a proof-of-concept example of how the predictive model could be used to inform a treatment decision. For this example, we consider two hypothetical depressed individuals, Patient X and Patient Y. Both patients have moderately severe depression with equal scores of 20 on the HRSD, 16 on the QIDS, and 15 on the PDSQ MDD scale. However, the patients were assigned different item-level endorsements as described below.

Patient X’s symptom profile on the HRSD and QIDS is one of extreme sadness and low self- worth with moderate anxiety, fatigue, indecision, and loss of libido and motivation. Patient X does not have early or late insomnia, but reports frequent and prolonged periods of wakefulness during the night. Patient X also reports mild appetite and weight loss, with little or no symptoms of agitation or psychomotor slowing. On the PDSQ, Patient X shows no evidence of co-morbid psychopathology and did not endorse any items indicating persistent depressive disorder.

Patient Y is similar to patient X in terms of fatigue, indecision, and motivation (though the latter is slightly worse for Patient Y), but differs in reporting little or no symptoms of sadness but severe psychomotor slowing, hypersomnia, agitation, anxiety, and loss of appetite and weight accompanied by moderate suicidality. On the PDSQ, Patient Y reports that her depression is highly persistent and also reports a high amount of comorbidity, including PTSD, OCD, GAD, panic disorder, agoraphobia, social phobia, somatization, hypochondria, and an eating disorder.

Patients X and Y differ starkly in their credulity regarding Deprexis, with Patient X expressing 100% confidence that the treatment can work and Patient Y expressing 0% confidence. Patient X also reports only mild functional impairment in her work, social, and family life, whereas Patient Y reports severe impairments in all three. Patient X is also from an economically disadvantaged rural area with extremely limited access to mental health providers (none within short driving distance), whereas Patient Y is from an affluent urban area where there is at least one mental health provider for every 100 residents who is within short driving distance.

For all other predictors (more than half), Patients X and Y were assigned identical values equivalent to the sample mean for numeric predictors and the sample mode for factors. To account for the potential impact of module usage (which we cannot know in advance), we further make 2 conditional predictions for each patient: one assuming low module usage (0 minutes for all modules) and one assuming high module usage (60 minutes for all modules). The following table gives the predicted scores and % improvement for each patient under each condition:

| Patient | Conditional usage | Pre-tx HRSD | Predicted Post-tx HRSD | % Improvement |
| --- | --- | --- | --- | --- |
| X | High | 20 | 4.8 | + 76 |
| X | Low | 20 | 6.9 | + 66 |
| Y | High | 20 | 20.8 | -4 |
| Y | Low | 20 | 23.3 | -17 |

This example illustrates that, despite the rather weak effects of each of these predictors on their own, they can all add up to a starkly different prediction for two individuals, even with identical scores on the most important single predictor, baseline depression severity. This also informs the treatment decision for the individual patients: for example, if there is only one slot available with a clinician, we would recommend in this case that it be given to Participant Y (who is predicted to not improve with Deprexis) over Participant X (who is predicted to achieve remission with Deprexis). This of course does not prove that Participant X would fare better with Deprexis over another treatment option (perhaps she is likely to get better regardless) or that Participant Y would fare worse (perhaps she would have deteriorated further without it). Such differential predictions could be obtained by training similar models to other treatment response data and then comparing the projected outcomes. We would also note that module usage appears to make about a 2.1-point difference in HRSD outcome for Patient X and a 2.6-point difference for Patient Y. This is interesting given that the mean HRSD outcome difference between those treated with Deprexis vs. those who were wait-listed was about twice this much (4.8 points) and suggests that just having access to the intervention is somewhat beneficial. With a much larger sample size, our hope is that it will be possible to more reliably capture interactions between baseline predictors and module usage patterns via the random forest or a similar algorithm that would enable us to make personalized recommendations about how much time an individual with a certain psychometric/demographic profile should allocate to specific modules to achieve the best therapeutic outcomes. However, based on this model, we would already recommend that certain modules—Relax, Relate, Cope, and Accept—be “front loaded” because they appear to benefit the average user the most.

In conclusion, we hope this example illustrates concrete actions that could be taken based on insights that this project has provided, but also how this can serve as a building block toward personalized psychological medicine.

References

Sheehan, D.V., Harnett-Sheehan, K. & Raj, B.A., 1996. The measurement of disability. *International Clinical Psychopharmacology*, 11, p.89.

Trajković, G. et al., 2011. Reliability of the Hamilton Rating Scale for Depression: A meta-analysis over a period of 49years. *Psychiatry Research*, 189(1), pp.1–9.

Watson, D. et al., 2008. Further validation of the IDAS: Evidence of convergent, discriminant, criterion, and incremental validity. *Psychological Assessment*, 20(3), pp.248–259.
